# Researchers’ perspectives on return of individual genetics results to research participants: a qualitative study

**DOI:** 10.1080/11287462.2021.1896453

**Published:** 2021-03-09

**Authors:** Erisa Sabakaki Mwaka, Deborah Ekusai Sebatta, Joseph Ochieng, Ian Guyton Munabi, Godfrey Bagenda, Deborah Ainembabazi, David Kaawa-Mafigiri

**Affiliations:** a College of Health Sciences, Makerere University, Kampala, Uganda; b College of Humanities and Social Sciences, Makerere University, Kampala, Uganda

**Keywords:** Researchers, genetics, return of results, perspective

## Abstract

Genetic results are usually not returned to research participants in Uganda despite their increased demand. We report on researchers’ perceptions and experiences of return of individual genetic research results. The study involved 15 in-depth interviews of investigators involved in genetics and/or genomic research. A thematic approach was used to interpret the results. The four themes that emerged from the data were the need for return of individual results including incidental findings, community engagement and the consenting process, implications and challenges to return of individual results. While researchers are willing to return clinically significant genetic results to research participants, they remain unsure of how this should be implemented. Suggestions to aid implementation of return of results included reconsenting of participants before receiving individual genetic results and increasing access to genetic counseling services. Community engagement to determine community perceptions and individual preferences for the return of results, and also prepare participants to safely receive results emerged as another way to support return of results. Researchers have a positive attitude toward the return of clinically significant genetic results to research participants. There is need to develop national guidance on genetic research and also build capacity for clinical genetics and genetic counseling.

## Introduction

Genome sequencing is increasingly being applied in research and clinical care, owing to the rapidly evolving technology, decreased costs and the reduced time needed to obtain results (Fiore & Goodman, 2016) (Foley et al., 2015). With the coming of the Human Heredity and Health in Africa (H3Africa) project (H3Africa, 2014), a lot of genetics and genomic research is currently being conducted on the African continent and researchers will soon have to return research results including incidental findings to participants and their families where indicated. Human Heredity and Health in Africa (H3Africa) is a consortium of researchers from Africa that was established in 2012. H3Africa aims to empower Africa researchers in genomic sciences, establish and nurture effective collaborative partnerships among African researchers based on the African continent and generate valuable data that could be used to improve global health (H3Africa). In the initial years of the H3Africa initiative, it was realized that most countries in sub-Saharan Africa had no national guidance for genetic research (de Vries et al., 2017). As a result, they have since developed several guidance documents on ethics, governance and resource sharing to foster best ethical practices within the consortium (H3Africa).

This paper discusses the issue of return of genetic results to research participants, a topic that has not been adequately explored in sub-Saharan Africa. Article 26 of the World Medical Association Declaration of Helsinki (WMA, 2013) recommends that research participants should be given the option of communicating feedback on the general outcomes of the study and results of the study. However, this is often not done for genetics and genomics research (Wallace & Kent, 2011)*.* Historically, large-scale longitudinal genetic and genomic research studies have not returned individual research results to their participants, as these studies are not intended to find clinically significant information for individuals (Wallace & Kent, 2011). However, this stance is now changing; commentators now argue that there is ethical imperative to return clinically significant results and individuals are now expressing a desire to have them (Bergner et al., 2014; Haga et al., 2011; McGowan et al., 2018; Pervola, 2018; Rutakumwa et al., 2019).

Safe return of results requires robust national guidelines, policies and regulations; however, most countries in Africa lack regulatory guidance on genetic research. Only three African countries have guidelines specific to genetic, genomic and biobanking research, although guidelines from seven countries make specific mention of the return of genetic results (de Vries et al., 2017). Furthermore, most African countries have no capacity to return individual research results because of insufficient numbers of qualified health professionals, poorly equipped institutions and lack of appropriate guidelines to guide decision making for feedback of research (Wonkam & de Vries, 2020). African countries, therefore, need to develop broad and flexible guidelines, policies and regulations for genetic and genomic research that can accommodate the fast evolving technologies (de Vries et al., 2017).

Genetic and genomic testing and research are relatively new to Uganda; and there is no national guidance on how this should be conducted and regulated. Currently most studies in Uganda do not return results and neither do research ethics committees (REC) emphasize the return of results. Most, if not all, health facilities, academic and research institutions have no professional genetic counselors and hence do not offer these services to patients and research participants and their families. Furthermore, there are neither laws nor local policies governing the return of genetic results. This study, therefore, aimed to explore researchers’ perceptions on the return of individual genetic results and how issues concerning return of these results should be handled during the informed consent process for genetic research to inform the development of ethical guidance and best practices for handling genetic results.

## Materials and methods

This qualitative exploratory study employed in-depth interviews for data collection. This study is part of a bigger ongoing study that is exploring the perceptions and experiences of various stakeholders on the informed consent process for genetic and genomic research in Uganda. For this paper, we present findings on researchers’ perceptions on the return of individual results and how return of individual results should be handled during the informed consent process for genetic research.

### Study setting

The study was conducted at Makerere University College of Health Sciences (MakCHS), one of the nine constituent colleges at Makerere University in Uganda. As the largest and most research intensive university in Uganda, Makerere University has tremendously impacted medical education and research capacity development in Uganda and East Africa through collaborative projects like Medical Education Partnership Initiative (MEPI) (Grant number: R24TW008886-05), Training Health Researchers into Vocational Excellence in East Africa (Grant number: 107742/Z/15/Z DEL-15-011), African Association for Health Professions Education and Research (Grant number: R25TW011217) and NURTURE: Research training and mentoring program for career development of faculty at Makerere University College of Health Sciences (Grant number: D43TW010132).

### Participants

All participants were researchers actively involved in genetic/genomic research in Uganda and affiliated to MakCHS. Participants were principal investigators of protocols involving host genetics/genomics that were approved by Uganda National Council for Science and Technology (UNCST) for 2012–2017. UNCST provides regulatory oversight of all research activities in the country; and per local regulations, all protocols approved by accredited research ethics committees are submitted to UNCST for approval and registration. We searched archived research protocols approved by UNCST for 2012–2017. Only investigators based at MakCHS and affiliate research institutes were eligible. A list of 23 investigators was generated and all were invited to participate in the study, but only 15 consented and participated in the study, of which three were H3Africa principal investigators. The number of researchers conducting genetics and genomic research at MakCHS is not known; however, it is important to note that there are several masters and PhD level scientists that are in training in genetic science and bioinformatics, mainly sponsored by the H3Africa initiative (H3Africa). There is only one clinical geneticist at MakCHS. To the best knowledge of the authors, there are no professional genetic counselors in the country, which emerged strongly as discussed in our findings.

### Data collection

Fifteen in-depth interviews were conducted between February and June 2019 by a team of four that included the principal investigator (ESM), two social scientists with experience in qualitative research methods (DES and DMK) and a research assistant (GB). The same team of four conducted all interviews to ensure consistency. Prior to the start of the study, the research team was trained on the protocol to ensure that they internalized and understood the study well. Data were collected using an in-depth interview guide that was developed by ESM, DMK and DES, and consisted of open-ended questions that explored researchers’ perceptions and experiences on the return of individual results and how issues concerning return of individual results should be handled during the informed consent process for genetic research. The interview guides were piloted and revised prior to the full data collection process.

All interviews were conducted in English, audio-recorded alongside detailed note taking, and later transcribed verbatim. On average, interviews lasted between 45 and 60 min. Debriefing meetings were held by the research team at the end of each interview to check on completeness and review preliminary perspectives that had arisen. All data were securely kept to ensure confidentiality.

### Data management and analysis

*V*erified transcripts were imported into NVivo 12 software (QSR International Pty Ltd, 2014) to manage and organize the data. Data analysis was conducted continuously throughout the study using a thematic approach (Braun & Clarke, 2006; Fereday & Muir-Cochrane, 2006). The first step of the analysis involved reading of all transcripts to familiarize, mark and memo the data. Three of the authors (DES, DKM, and ESM) then developed a codebook. They then independently developed codes by performing open line-by-line coding to generate the first set of codes. The codebook was then refined to identify themes in relation to participants’ perspectives and experiences on return of individual genomic results. A thematic approach (Braun & Clarke, 2006) was used to generate emergent themes and interpret the results and comparisons were made. Where differences emerged among the independent coders, they were solved by consensus. Findings were supported by representative quotes.

### Ethics approval

Ethics approval was obtained from the Makerere University School of Biomedical Sciences Higher Degrees and Research Ethics Committee (SBSHD-REC 517) followed by clearance by Uganda National Council for Science and Technology (SS 4490). Written informed consent was obtained from all participants prior to interview. Data were kept securely, and all recordings and transcripts were de-identified, assigned special codes and stored on a password-protected computer. No participant identifying information was published.

## Results

Fifteen individuals participated in the interviews. The majority was male (12/15) with a mean age of 42.8 years (Range: 29–56 years). All interviewees were Ugandans involved in international collaborative research. Five of the interviewees were clinical researchers, six were clinical epidemiologists and three were basic genetic scientists. Only one interviewee had formal training in clinical genetics, and only three were investigators on research projects where genetics was a primary objective. Participants had, on average, participated in research for 12 years (SD 1.2, range: 3–22 years). The socio-demographic information is summarized in Table 1.

**Table 1.** Demographic information.

*Gender*	
Male	12
Female	3
*Highest level of education*	
Masters	6
PhD	9
Role in research	
Clinical researcher	5
Basic scientist	3
Clinical epidemiologist	6
Clinical geneticist	1
*Research experience (years)*	
0–5	2
6–10	3
11–15	8
>15	2

There were four emergent themes including (1) the need for return of individual results including incidental findings, (2) community engagement and the consenting process, (3) Implications of return of results, (4) challenges to the return of individual results (Table 2).

**Table 2.** Emergent themes.

Theme	Key findings
The need for return of individual results including incidental finding	Positive attitudes toward return of results but only clinically proven and validated results should be returned. Genetic counselors were seen as vital in the return of results however there are no professional genetic counselors in the country. Incidental findings of clinical significance should be returned but this should be appropriately done to prevent harm to individual participants, families and communities. REC approval should be obtained prior to returning results.
Community engagement and the Informed consent process	Investigators, REC members and the general public poorly understand genetic/genomic research. Community engagement should be done to understand research participant preferences, identify the most appropriate strategies for returning results and also prepare communities to receive the results. Research participants should be given adequate information on the implications of returning results and ensure that they understand. Research participants should be re-contacted and re-consented before any results are returned.
Implications of return of results	Return of genetic/genomic results is sensitive and should be handled appropriately. Breach of confidentiality could result in risks of harm to the individual participant, their families and the community. Societal misconceptions of genetic research could result in community misinterpretation of research results. Mishandling of results could impact on future research activities in the affected communities.
Challenges to the return of results	Most genetic findings are abstract and have limited clinical utility. There are logistical challenges in tracing and recontacting research participants. There is inadequate technology and limited local capacity to analyze and interpret results. Challenges in dealing with research participants’ expectations.

### The need for return of individual results to research participants, including incidental findings

Overall, interviewees had a positive attitude toward the return of clinically significant individual results to research participants, although they all reported that they had never participated in returning genetic results to research participants. They felt that the return of genetic results, including incidental findings to research participants, is complex; particularly in low resourced settings. They asserted that it is the investigators’ obligation to return individual results of genetic testing; however, they emphasized that research ethics committee (REC) approval of the results should be first obtained. They also argued that only proven and validated genetic results should be returned after genetic counselling.

… If you’re doing your lab analyses and you’re [analyzing] these genes and maybe you see something that will benefit the patient clinically; for example, if it is already approved and this variation in the gene may increase one’s risk of getting this disease and the patient will benefit from that finding. Then I think it’s important to report back and seek medical attention, only if such a finding is already approved. (R14, Female)

Most interviewees expressed dismay at the absence of professional genetic counsellors in the country, just like it is in most of sub-Saharan Africa. They appreciated the role of genetic counsellors whom they thought were important in routine clinical care and research. Furthermore, genetic counsellors were perceived as having sufficient knowledge on genetics, genetic testing and result interpretation, and counseling experience. Genetic counselors were seen as key in ensuring that research participants understand genetic research and its implications.

I would think so, because as I explained at the beginning, the challenge we have is that trying to translate this down to the simplest level of understanding is a challenge. It’s a challenge given that our communities have varying levels of education and therefore varying levels of comprehension. So, if we have a specialized person, more specialized people tend to understand this more in detail than none specialized people. (R10, Male)

Another interviewee said

I have previously told the H3Africa Consortium that the need is not to train more geneticists. The need is to train more genetic counselors. (R12, Male)

Whereas the majority of interviewees felt that it was the investigator's obligation to convey research results to study participants, some advised caution when handling genetic results. They pointed out that a lot of the genetic and genomic results are not validated and are of unknown utility and as such, should not be communicated to research participants.

I will tell you that there are many variants we are [currently] identifying in the DNA that diverge from the normal and we call them variants of unknown significance (VUS) because we don’t know what they are associated with. Are they associated with risk to heart attack? Who would want to know a variant of unknown origin? (R12, Male)

There is a lot of debate on how secondary findings of genetic testing should be handled. When asked for their opinions on the return of incidental findings, a majority opined that results of clinical significance should be returned to participants.

One interviewee observed the need to return clinically significant results

… Really if something is of clinical importance and you think disclosing such information will be helpful or it will help in the management of the participant, I think such information should be disclosed. Otherwise why withhold it if it will be of benefit to the person? But if it will not be of benefit then [withhold it] because there are other challenges, how will you again re-contact the person and so on? It is a bit challenging. If something is of extreme importance, I still think efforts should be made to give such a feedback. (R07, Male)

Others thought that incidental findings should be considered on a case-by-case basis; however, interviewees underscored the importance of preparing patients for such results during the informed consent process. Some interviewees indicated that some of these finding could potentially involve more than the individual participant; and advised that extra precautions be taken to reduce the risk of emotional pain; psychological risks like depression; social harm to individual participants and their families; and stigma and discrimination of communities. They indicated that all these have to be taken into consideration before deciding whether to return such findings or not.

This genetics you know can bring you information that you never intended or you were never prepared to receive. I mean they can easily tell you that you might get multiple sclerosis according to what we see, and multiple sclerosis means that you are not going to live this long and that might not be for only you. It might be for your relatives so those are the type of things this type of research brings in and as you have said for example of course I will answer many things. (R09, Male)

Interviewees also felt that RECs should decide which results should be returned and when they should be returned.

Let the IRB [Institutional review board] decide whether they give it [results] to the patient or not because the IRB is literary the eyes and ears [of the participant]. (R12, Male)

### Community engagement (CE) and the informed consent process

Informed consent is an ethical imperative in research involving humans as participants. Interviewees felt that some investigators, REC members and the general public poorly understand genetic research. They highlighted the need for community engagement to prepare participants to receive genetic results, both individual and aggregate results. They further emphasized that this should put into consideration the potential risks of returning the results.

So, in other words, personally I do believe that information should be returned to the participant but there should probably also be an issue of a community intervention because genetics is far beyond one individual. Somehow maybe there could be community engagement to understand what could be the implication and also be involved in this disclosure and consenting process because these things affect them in one way or another. (R09, Male)

Interviewees opined that community engagement should be a continuous process from protocol development, study implementation to result dissemination so as to determine the most appropriate strategies for returning genetic results.

But in terms of engaging the community or study participants I think the key thing is that uh there is engagement on the individual level in which case for instance you are consenting a particular patient. But there is engagement also at the community level in which case you are trying to inform the larger community. If am to talk about the engagement at a community level, CAFGEN has been very much engaged in community outreach, uh we developed a book; it’s actually a comic book. (R12, Male)

Interviewees also indicated that, it is imperative that research participant preferences on the return of results are sought during the consenting process. They asserted that research participants’ preferences should be respected and taken into consideration.

… But it also depends on what specifically that particular research has set out to do … . they may not find anything because they have set to find risks, but if and when they do, I think it is only right to give the participant the opportunity to make the decision as to whether they want to know, or do not want to know the findings and having given them all the information and the potential implications of what has been found. (R11, Male)

The importance of giving participants adequate information was emphasized so that they not only understand what the study is all about but also appreciate the implications of returning results.

… Because I totally think and believe knowledge is power although it doesn’t mean everything because I give you a scenario like if a person knew that if they get pregnant there’s a 50% chance of their child having a congenital abnormality … (R09, Male)

Interviewees were asked how best the return of individual results should safely be communicated to participants. Several researchers preferred that research participants be traced and re-consented before any genetic results are communicated to them. One researcher said

I think you do it in two ways, one you could cover yourself within the consent and make the participant aware that “in case something happens, we may come back to you” and consent at that point in time. The other way is, if it is not covered from the start of the consent, again you can approach the participant and let them know that this is what we are finding and if they agree or not agree I think that has to be documented too at that point in time so that from the researcher point of view you are also protected that you did not hide any information and also from the participants side there is evidence that you provided this and it was their own choice to agree or not to agree to the information. (R11, Male)

However, one researcher emphasized that participants should only be re-contacted when the results are of direct benefit to them

But if it is not of benefit then … how will you again re-contact the person and so on? It is a bit challenging. (R08, Male)

### Implications of return of results

The return of genetic and genomic results is associated with a great deal of societal implications. Interviewees pointed out that the return of genetic results is a sensitive issue that if not handled appropriately could potentially impact negatively on individual participants, their families and entire communities. They noted that these results could lead to disharmony and disputes in the family especially if there is breach of confidentiality.

If you are revealing results for example, if you call up a patient and you say these are the results of your genetic profile, if it has implications to a family in terms of … , you have a disease that is not curable and people from the family, your spouse, your mother, your children finding out so it might have that risk of informing people who shouldn’t know. Who might feel that the information is more harmful to them and destructive. (R15, Female)

Research findings could also culminate in harm to the wider community.

And so ahh, yeah what are people comfortable with if it becomes stigmatizing to them as a community? As I said, I guess the classic example is if you publish something about a community in terms of its genetic risk to disease. You know people may not want to go and get husbands or wives from there. So, I think it’s more of a community stigma if you find something that makes them stigmatized especially if its disease related. (R06, Male)

Interviewees also noted that many local communities are highly superstitious and believe in sorcery and witchcraft. They, therefore, recommended engaging the community to dispel some of these beliefs and avoid misperceptions of biobanking and the misinterpretation of research results.

So the experience of community engagement if am to define, I have engaged because when you restrict it to participants then you are looking at study subjects, but I have engaged the community [before] in terms of genetics at different levels and not just in context of studies but also in context of you know social issues. I was involved in trying to advise the government on the issues of homosexuality. A series of people have presented the argument that homosexuality is a genetic thing and of course there was need to try to educate people about the genetics of the inheritance of homosexuality. (R12, Male)

Implications to future research activities were also raised. Participants pointed out that a breach in confidentiality or mishandling of research results that lead to the identification of individuals and communities could potentially have far-reaching implications for future research activities

Ahh if they find information, which may not be good about somebody and the person is identified maybe by name, by place and if they are talking about people who come from this place, they had such findings. You know it may influence the community if initially you had told them things would be anonymized and that is breached. When they get such information, next time they may not have interest in participating in the study in future studies and also in most cases you see that these things may be work done together with the collaborators and so it may risk such collaborations. So generally, it is not conducive for further conduct of research in the community. (R08 Male)

### Challenges to the return of results

Interviewees highlighted several challenges to the return of individual results. They indicated that to the best of their knowledge, no study in Uganda was returning genetic results; and several of these studies explicitly state in the protocol that they would not return results. Interviewees attributed this to: most genetic results not being of clinical importance; the long time it takes to analyze and interpret genetic data; and logistical challenges in tracing and re-contacting research participants.

It’s not easy, it’s a very difficult process because it’s not fast as you said, many times results will come out like six years after, and so it’s going to be costly to go back and look for these people and then explain to them about these coming the results. (R09 Male)

Interviewees noted that there is inadequate technology and limited local capacity to analyze and interpret genetic data. They also noted that there are no well-defined platforms to discuss the return of genetic results.

Yeah but you see with the available technology you cannot use that information even if you got it. Even if as a PI [principal investigator] now I got the full sequencing of my genome, I cannot not use it for anything because the technology that we have is not supportive of utilizing that information. … So genetic data is hardly useful, you know it’s not like a cognitive test or an eye test or knowing whether you are depressed or not. (R02 Male)

Interviewees also observed that the field of genomics is fast evolving and thus the need for local scientists to keep pace. They pointed out that some genetic investigators do not have adequate knowledge of genetics and this could affect their capacity to accurately interpret genetic results. One male investigator observed

Partly yes but it is a learning process particularly for genetic and genomic research, the researchers are also learning. Like we said in the beginning, these concepts are new, you find even NIH is just defining them. So, to me, it wouldn’t surprise me in case Ugandan scientists also are learning. (R07 Male)

He further said:

NIH [National Institutes of Health] has done it, America has done it, we also need to come to agreeable terms of what genomics or genetics research is. That is only when we will reduce on the misunderstanding of this concept but for me I expect it because these are new sciences and I don’t blame the Ugandan Scientists or even the Ethics Committee for not understanding it, these are just new things. (R07, Male)

One investigator observed that there may be varying interpretation of same results depending on the type of analysis; and this in her opinion makes it difficult for such results to be communicated to participants:

There is a finding here, should we disturb you all over again and tell you there is a finding here? How many times are we going to look you up and tell you? Because different people have used different [methods] in the whole world [to analyze] the same genome. (R04, Female)

At the individual level, respondents noted that people have different motivations for participating in research. Many individuals, especially in low resourced settings are usually motivated by the need to have their health issues addressed. Interviewees also pointed out that most clinical studies usually return results of routine laboratory tests, but this was not the case with genomic/genetic studies. Some interviewees reported that they often face challenges in dealing with research participants’ expectations in this regard.

… the other challenge, people keep on asking you about the results because I think our society is used to … they take off your sample, you get your results and then you use your results. Now to tell people that they are not going to get these results themselves, or that even if they got the results, it might be difficult to use them in the current state of technology, it’s difficult. It’s a challenge. (R02, Male)

Concerning the actual return of results, Interviewees recommended the phased release of genetic results as a way of preventing social harm to individuals and communities

I think that if the results came out but they (participants) had not requested for them, but the results have public health significance, I think it is worthwhile giving them in generality first, and waiting to see if that has attracted interest from people. (R13 Male)

The same interviewee added:

So you have to inform the ministry of health, the local government especially the district health offices and then this information should in general terms be passed on to people so that if it possible, some of those things can be dealt with. (R13 Male)

## Discussion

In Uganda, just like in much of sub-Saharan Africa, genetics and genomics results are generally not returned despite the increased demand for them (Rutakumwa et al., 2019). Research participants are increasingly becoming frustrated by the lack of feedback from researchers and the non-return of genetic results; and this has been reported to negatively affect community interest in participating in research (Tindana et al., 2020).

Overall, interviewees in this study had a positive attitude toward returning individual genetic research results, and recommended that clinically significant results including incidental findings should be returned to research participants. However, for this to happen, there is a need for clear national guidelines to ensure that individual participants and their communities are adequately protected. While there is no legal obligation for researchers to return results (Roberts et al., 2017), it is considered an ethical requirement for the researcher to return results (Edwards et al., 2018). Chapter 11 of the Ugandan ethics guidelines states, “Researchers shall, as appropriate, make all reasonable efforts to share findings of research with the host organization, research participants, key stakeholders and communities in which research was done” (UNCST, 2014, p. 32). Giving feedback is also recommended in article 26 of the Declaration of Helsinki (WMA, 2013). But, both documents do not give any specific guidance on how genetic results should be handled. Interviewees had varied opinions on the return of individual results, suggesting that a “one size fits all” approach would not be appropriate (Stein et al., 2019; Vos et al., 2017). Investigators with a positive attitude toward the return of genetic results argued that only validated clinically significant individual results should be returned to the research participants (Dreyfus & Sobel, 2018; Seidman et al., 2017). However, these results have to be clinically validated by a certified/accredited laboratory (H3Africa, 2018; Holm et al., 2014) and require scientists with the capacity to accurately analyze and interpret them. Just like elsewhere in Africa, this capacity is lacking; there is shortage of medical genetic professionals with the competence to interpret and unravel the significance of genetic results and translating them in meaningful and comprehensible way to research participants (Wonkam & de Vries, 2020).

Research participants expect feedback on the progress of the study and any available results (Gaieski et al., 2019; McGowan et al., 2018; Tindana et al., 2020). They have a right to their results; however, they too have preferences in regard to return of results that should be sought and respected during the consenting process (Gornick et al., 2017; Kullo et al., 2018; Ryan et al., 2017). This helps researchers determine whether participants want to receive their results or not; which results they want to receive; and the preferred mode of communication (Kaphingst et al., 2016; Middleton et al., 2016). However, when considering this, investigators should carefully gauge research participants’ expectations, some of which may not be feasible. Interviewees asserted that research participants should, therefore, be prepared to receive these results during the informed consent process. They also stressed that participants should be informed about the possibility of returning results; and their decisional preferences for the return of individual results should, therefore, be sought. In the absence of robust ethical guideline for genetic research in much of sub-Saharan Africa, Uganda inclusive, reference is made to the H3Africa ethics, governance and resource sharing documents (H3Africa). Relevant to this study is the guideline on informed consent for biobanking and genetic research (H3Africa, 2017); and the return of individual genetic research findings to participants (H3Africa, 2018). These guidelines acknowledge the complexities associated with the return of individual genomic study results in Africa and propose a decisional tree that takes cognizance of the ethical and social implications of returning these results. All interviewees indicated that they had no experience in returning individual genetic results to participants, and for most of them; genetics is usually not the primary objective in their research projects. However, they had clear understanding of what return of individual results entails, and a majority acknowledged the importance of genetic counseling.

Concern was also raised on the absence of genetic counselors in Uganda. While the role of genetic counselors is vital in ensuring that research participants understand genetic research and its implications, there are no professional genetic counselors in much of sub-Saharan Africa (Hooker et al., 2017; Mboowa & Sserwadda, 2019; Reuter et al., 2018; Roberts et al., 2017). Genetic counseling as a profession has been in existence for more than 50 years in high-income countries; however, it has failed to grow, particularly in low resourced settings where it is not appreciated and genetic counselors may not be seen as essential, and are looked at as an avoidable cost (Biesecker, 2018; Roberts et al., 2017). As of 2016, there was only one formal genetic counseling services and training program, and less than 20 genetic counselors on the entire African continent (Abacan et al., 2019). As one principal investigator indicated … the need is not to train more geneticists. The need is to train more genetic counselors. African countries, therefore, need to invest in training this cadre of healthcare providers whose role is increasingly becoming essential with the global trend toward precision medicine. The H3Africa Consortium is trying to address this challenge through online training in genetic counseling for nurses across Africa through the African Genomic Medicine Training Initiative (AGMT) (Mulder et al., 2019)**.**

Views on community engagement and the informed consent process emerged prominently in this study. Community engagement is a key ethical requirement in genetic and genomic research (Faure et al., 2020; Moodley & Beyer, 2019; Staunton et al., 2018; Tindana et al., 2017; Tindana et al., 2015). The Uganda national ethics guidelines also recognize this and recommend community engagement “right from the inception of research to post research period” (UNCST, 2014). Valid informed consent demands that the participant makes a voluntary decision after adequately understanding relevant information concerning the study. However, interviewees observed that local investigators, RECs and the general public have limited understanding of genetics; but this is not unique to Uganda only (Ogunrin et al., 2019; Stein et al., 2019; van Wyk et al., 2016; Vos et al., 2017). Because of this, authors have proposed community engagement models that aim to optimize informed consent processes (Beaton et al., 2017; Moodley & Beyer, 2019; Tindana et al., 2017; Tindana et al., 2015). Community engagement is very important in enhancing genetic education of the public and the understanding of the ethical, legal and societal implication of this field of research. Community engagement also facilitates researchers’ understanding of community perspectives, beliefs, practices and preferences (Tindana et al., 2017).

Another important theme from our data was the implications of genetic results on individual participants, their families and the wider community. There exist several cultural differences in our society from rural communities adhering to long-established cultural belief systems and practices on the one hand, to the educated people residing mainly in urban areas (H3Africa, 2014). Many local communities are highly superstitious and suspicious of any studies that involve the collection and storage of human tissues; and this may influence research participants’ attitudes and preferences in regard to the return of individual results. The H3Africa informed consent guideline (H3Africa, 2017) acknowledges the complexities associated with the return of individual genomic study results. The guideline recommends that participants should be informed whether their individual results will be returned or not. Careful consideration should be made on the implication of these results on the individual, family and wider community. The Ugandan ethics guidelines also reiterate the need to be sensitive about the ethical implications of returning of research results. The guidelines further advise that researchers put in place appropriate measures to protect research participants and their communities (UNCST, 2014). Communities should, therefore, be adequately prepared to receive these results through community engagement (Beaton et al., 2017; Faucett & Davis, 2016; Zusevics et al., 2017). This entails the dispelling of some of these beliefs and avoiding misperceptions and misinterpretation of genetic results. Concern was also raised on the implications of breach of confidentiality on the trust between the community and research teams. Trust is very vital to the success of research projects; it not only facilitates recruitment and retention of participants but also impacts future research activities in the affected communities (Staunton et al., 2018).

One of the major challenges to return of individual results identified in this study was the general poor understanding of genetics-related information by various stakeholders. Majority of the understanding is limited to issues of paternity and the fact that genetics is a relatively new area in Africa (Dick et al., 2017; Kengne-Ouafo et al., 2016). Furthermore, there is no specific national guidance on how exactly these results should be fed back to participants. A majority of interviewees preferred the tracing and re-consenting of research participants if any genetic research results were to be returned. This finding concurs with several studies that indicate that research participants prefer being re-consented (Condit et al., 2016; Dixon-Woods et al., 2017; Edwards et al., 2016; Sutton et al., 2019). However, interviewees noted that genetic results (if any) come back after a long period and the process of delivering them to participants is expensive and is associated with logistical and technological challenges that local scientists may fail to address (Middleton et al., 2016; Stein et al., 2019).

### Study limitations

The study recruited study participants from a single institution (MakCHS). However, we believe their views are representative of the wider scientific community in Uganda because of the impact MakCHS has on academia and the research enterprise in Uganda and the East African region. Additionally any other institution engaging in genetic research would likely face similar contexts as at MakCHS. We also did not obtain detailed information on interviewees’ training in clinical genetics as such it was difficult to compare their responses depending on specific training in genetics. Our study did not elicit discussion of return of results in a pediatric setting because no specific intent was undertaken to review these themes for pediatric settings.

## Conclusion

There is an increasing demand for the return of individual genetic research results; however; there is no national guidance on how this issue should be handled. Our study suggests that genetic researchers have a positive attitude toward the return of clinically significant genetic results to research participants. However, they seem to be unsure of how this should be implemented because of lack of national guidance and genetic counseling services. Return of genetic results has ethical and societal implications that if not handled appropriately could potentially impact negatively on individual participants, their families, communities and future research activities in affected communities. Therefore, there is a need for community engagement to determine individual participant and community perceptions, and preferences for the return of results, and also prepare participants to safely receive these results. There is a need to develop locally appropriate ethical guidance and best practices for handling genetic results. We further encourage research to determine the most ideal strategies for returning individual genetic results in these settings.

The following recommendations can be drawn from our findings:

First, robust national guidelines for conducting genetic research in Uganda should be developed. The guideline development process should involve wide stakeholder engagement and should also be guided by relevant local research. Therefore, more empirical research involving various stakeholders should be conducted to generate a strong evidence base to inform guideline development. These guidelines should also be bench marked on the existing internationally accepted guidance documents from entities such as the H3Africa Consortium (H3Africa), National Academies of Sciences and Medicine (2018), American College of Medical Genetics and Genomics (Kalia et al., 2017) to mention a few.

Second, there should also be capacity building for clinical genetics. Over the last decade, the H3Africa Consortium has been building capacity for genomic science and bioinformatics in Africa. However, inadequate focus has been put on training clinical geneticists and genetic counselors. Therefore, there is an urgent need for training this cadre of health professionals to create a critical mass of specialists who can safely return genetic and genomic results to patients in clinical care and research. For a start, nurses should be encouraged to undertake basic online training in genetic counseling under the African Genomic Medicine Training Initiative (AGMT) (Mulder et al., 2019).

Finally, community engagement activities should be scaled up to prepare communities for the return of genetic research results as and when they are available. Community engagement will help establish people’s expectations and preferences for the return of results. In addition, community engagement will enable researchers to learn more and appreciate community perspectives, beliefs and practices that could impact the return of results.
